# Review: Interventional radiology in peripheral vascular disease

**DOI:** 10.4103/0971-3026.40301

**Published:** 2008-05

**Authors:** Mathew P Cherian, Pankaj Mehta, Tejas M Kalyanpur, Prashanth Gupta

**Affiliations:** Department of Radiology, Kovai Medical Center and Hospital, Avinashi Road, Coimbatore, Tamil Nadu, India

**Keywords:** Angioplasty, embolization, interventional radiology, PVD

## Abstract

Peripheral vascular diseases (PVD) are referred to as diseases affecting the blood vessels other than the heart and the brain. Interventional endovascular treatment whenever feasible has become the first line of management in the treatment of PVD. Interventions may be aimed at either revascularization or deliberate occlusion of a diseased vessel(s). This article reviews the various peripheral vascular diseases with their appropriate endovascular management.

The history of interventional radiology (IR) dates back to 1953 when Seldinger published his article that described his technique of percutaneously accessing the blood vessels. It was Charles Dotter who coined the term “Interventional Radiology” on 10 June 1963 at the Czech Radiology Congress. He finished with a historic conclusion, which laid the foundation for IR: “The angiographic catheter can be more than a tool for passive means for diagnostic observation and used with imagination it can become an important surgical instrument”.[1] Dotter made history on 16 January 1964 with the first ever percutaneous transluminal angioplasty (PTA) of a stenotic femoral artery. Joseph Rosch developed the technique of transjugular intrahepatic portosystemic shunt in the late 1960's. He also developed embolization techniques for the treatment of gastrointestinal (GI) bleeding, transjugular liver biopsy and fallopian tube catheterization.

Cesare Gianturco was fundamental to the development of “coiled springs” used for arterial occlusion, the bird's nest vena caval filter and biliary and vascular stents. Andreas Gruentzig's invention of the double lumen catheter for balloon angioplasty in 1975 set in motion another revolution in the management of vascular diseases. Julio Palmaz was the first to develop a balloon expandable stent, which continues to be the cornerstone of coronary and renal revascularization. Intervention in PVD can be broadly classified into: (a) techniques for revascularization and (b) techniques resulting in vascular occlusion.

## Techniques for Revascularization

Occlusive disease in the blood vessels of the lower limbs classically presents with features of ischemia, which in the early stages manifests as pain during exertion of the affected limb (claudication). It then progresses to rest pain and finally ulceration, gangrene and limb loss.

The various endovascular methods for revascularization in PVD include percutaneous transluminal balloon angioplasty (PTA) and stenting, intra-arterial thrombolysis, thrombectomy, atherectomy, ultrasound angioplasty, laser angioplasty, cryoplasty, radiation and gene transfer therapy.

### Percutaneous transluminal angioplasty and stenting

**Figure 1:**
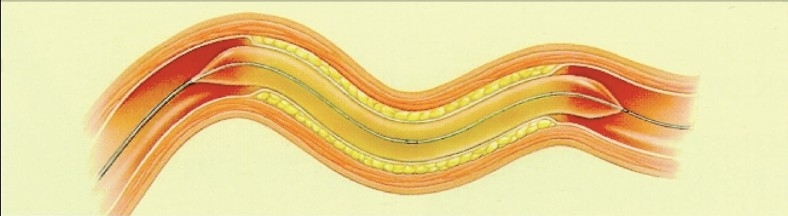
Angioplasty balloon catheter in its inflated state

PTA is performed predominantly with balloon catheters. The lesion is crossed with a guide wire and a balloon catheter is tracked over the wire and positioned across the lesion. On inflation and deflation, the occlusion opens secondary to plaque fracture, intimal separation and stretching of the media [Figure 1]. A vessel may not respond to angioplasty as a result of acute elastic recoil or due to a flow-limiting sub-intimal flap, both of which can be treated effectively by deploying a metal tubular mesh (stent). In specific locations like the renal ostia and the origins of the internal carotid arteries, elective stenting improves the patency rates.

The best results of PTA are achieved in stenotic lesions that are short, concentric and noncalcific. Cryoplasty, coated balloons, new-generation stents and stent grafts show promise in preventing restenosis.

Stents are classified as balloon and self-expandable. Balloon-expandable stents are crimped on a balloon and deployed during inflation. The single greatest advantage of these stents is their ability to be deployed precisely at the site of stenosis. Self-expanding stents, on the other hand, have an inherent memory instilled during the manufacturing process to expand to a predetermined diameter and are flexible. They are commonly used in all major peripheral vessels.

Covered stents represent a separate class of stents and have a polytetrafluoroethylene (PTFE) covering. These devices are predominantly used in applications such as the treatment of aneurysms, pseudoaneurysms, traumatic arterial perforations and arteriovenous fistulas (AVFs). Their role in cases of complete occlusion is being evaluated. Drug-eluting stents are the latest in stent technology aimed at overcoming the problems of in-stent restenosis. The ones that are commonly used are coated with paclitaxel and sirolimus. Although their efficacy in the coronary arteries has been proven,[2,3] their role in the peripheral arteries has not shown promise. Biodegradable stents were developed first by Stack *et al.* at Duke University.[4] They retain their radial strength for 1 month and are almost completely degraded by 9 months. Tamai *et al.*[5] first reported on the immediate and 6 months' results after the implantation of a biodegradable poly-L-lactide (PLLA) stent in humans. This type of biodegradable stent is not associated with intimal hyperplasia as in stainless steel stents.

### Endovascular therapy of carotid disease [Figure 2]

**Figure 2 (A, B):**
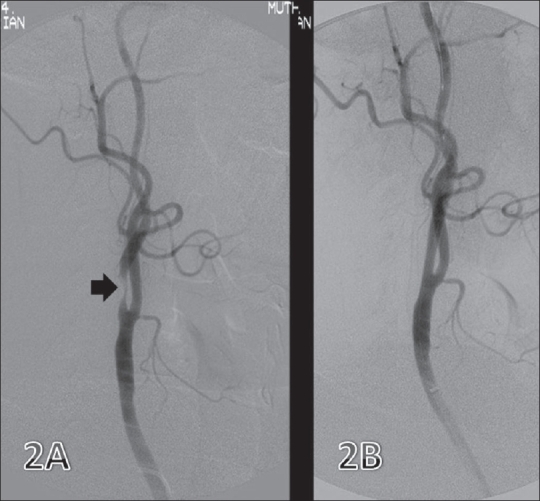
Internal carotid artery (ICA) stenosis. Preintervention image (A) shows a short segment stenosis (arrow) in the proximal ICA. Post-stenting image (B) shows good flow through the diseased segment

The Carotid and Vertebral Artery Transluminal Angioplasty Study trial (CAVATAS),[6] a prospective randomized study, compared carotid endarterectomy and PTA of the extracranial carotid artery. The study found that there was no difference between the results of the two methods over a period of 4 years. Stent-supported carotid angioplasty (SSCA) has reduced the risk of neurologic complications that occur with PTA for extracranial carotid artery stenosis. The preliminary results of SSCA[7–10] are encouraging, but no randomized trial has been conducted yet. The perioperative results of 307 patients randomized in the Stenting and Angioplasty with Protection in Patients at High Risk for Endarterectomy (SAPPHIRE) trial showed that the perioperative stroke and death rates were 4.4% for patients treated with angioplasty and stenting and 7.3% for those undergoing endarterectomy.[11] At present, scientists are in agreement that carotid angioplasty is an acceptable alternative to surgery.

### Endovascular treatment of renal artery stenosis [Figure 3]

**Figure 3 (A, B):**
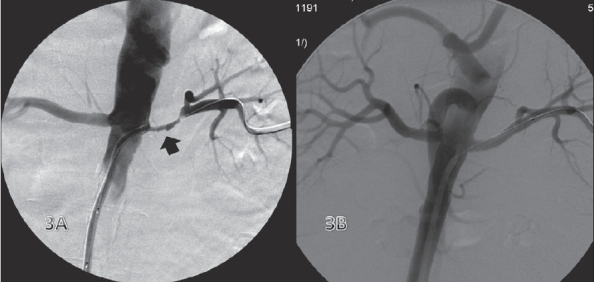
Left main renal artery stenosis. Preintervention image (A) shows stenosis of the left main renal artery (arrow). Post-stent image (B) shows good flow and caliber

PTA is accepted as the treatment of choice for renovascular hypertension caused by fibromuscular dysplasia (FMD) and nonostial atheromatous lesions of the renal artery.[12] Renal artery stent placement was described by Palmaz *et al.* as early as 1987.[13] In a prospective randomized control trial comparing PTA alone with PTA plus stent placement for ostial lesions, Van de Ven[14] and associates reported restenosis at 6 months in 14% of patients with stents, while those with only PTA had a restenosis rate of 48%.

### Endovascular treatment of lower limb occlusive disease

At present, the primary indication for intervention in lower limb arterial disease is a patient who has symptomatic arterial disease not adequately managed medically. When considering PTA, the lower extremity arterial system can be divided into three distinct anatomic territories: Aortoiliac, Femoropopliteal and Infrapopliteal.

#### Iliac arteries:

Iliac arteries are best treated by endovascular therapy. Successful angioplasty improves the blood flow in the vessel and increases flow to the collaterals in patients with associated femoropopliteal disease, thus leading to better long-term results especially when multiple segments are affected in the same leg.[11] Iliac angioplasty is useful not only for dilation of the primary lesion but also as an adjunct to definitive femoropopliteal surgery. In the Toronto series, 667 iliac percutaneous transluminal angioplasties were analyzed. At one month after angioplasty, 90.2% of the procedures were considered a success. Late success rates were 75.2 ± 1.8% at one year, 64.9 ± 2.1% at two years, 59.7 ± 2.2% at three years, 56.7 ± 2.4% at four years and 53.4 ± 2.7% at five years.[15]

#### Femoropopliteal disease [Figure 4]:

**Figure 4 (A, B):**
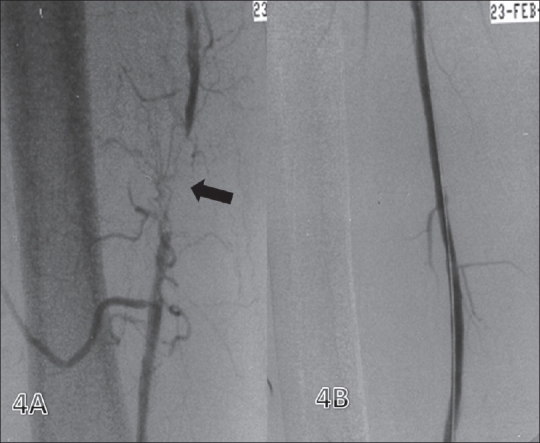
Superficial femoral artery (SFA) stenosis. Preintervention image (A) shows a short-segment mid-SFA occlusion (arrow) with reformation distally. Post-angioplasty (B) image shows good caliber and flow

Atherosclerotic involvement of the femoropopliteal segment is the most common cause of claudication.[16] The femoropopliteal segment differs from the iliac artery in that it is smaller, has a higher resistance and is more susceptible to spasm; it also has a lower blood flow rate. These factors explain the higher incidence of restenosis following PTA.[11] The recent ACC/AHA guidelines on peripheral arterial disease management do not recommend primary stent placement in the femoral, popliteal or tibial arteries. Provisional stent use (in which stent placement is limited to cases of suboptimal PTA results), however, remains an acceptable alternative. This may change in future with the availability of dedicated femoral stents with very low fracture rates. In the University of Toronto series, 254 PTAs of the femoral or popliteal arteries were performed in 236 patients. The procedures were successful in 96% of patients. When evaluated 1 month after the procedure, 88.8% of the procedures were considered successful (both the clinical grade and the noninvasive vascular laboratory measurements improved). Late success rates were 62.5 ± 3.2% at 1 year, 52.6 ± 3.5% at 2 years, 50.7 ± 3.5% at 3 years, 44.1 ± 4.0% at 4 years, 38.1 ± 4.4% at 5 years and 35.7 ± 4.8% at 6 years.[15]

#### Infrapopliteal disease [Figure 5]:

**Figure 5 (A, B):**
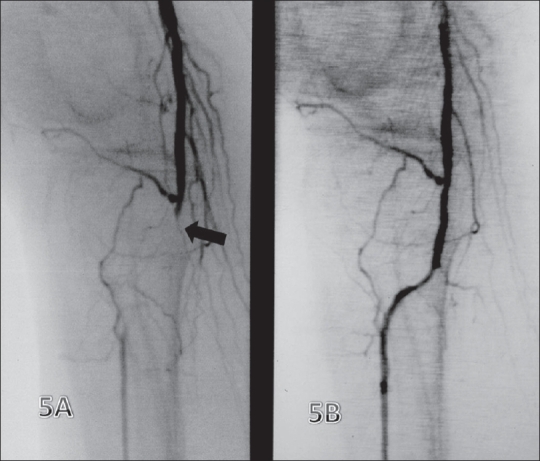
Infrapopliteal disease. Preintervention image (A) shows occlusion (arrow) of the proximal anterior tibial artery (ATA) with nonvisualization of the popliteal trifurcation with reformation (arrowhead) seen at the level of the mid-ATA. The post-angioplasty image (B) shows no residual stenosis in the ATA

Infrapopliteal PTA is primarily offered to patients with critical limb ischemia or lifestyle-modifying claudication.[15] Infrapopliteal PTA is also used to improve the results of proximal revascularization procedures, such as femoropopliteal PTA or bypass surgery in cases where poor outflow threatens graft patency. In our (as yet unpublished) series of 197 lesions, the two-year limb salvage rate was 83%.

At present, though no definite guidelines are available, a few points should be kept in mind when considering infrapopliteal angioplasty. Despite the high incidence of restenosis, temporarily restoring flow to one infrapopliteal artery allows ischemic ulcers to heal and sometimes rest pain to decrease.[11] Claudication alone is rarely an indication for infrapopliteal PTA. Stents have a very small role to play in these vessels, although the initial clinical experience with the 4-F self-expanding XPERT Stent System for below-knee disease shows promise and may be an answer to suboptimal angioplasty in this region.[17]

### Complications of angioplasty

Complications[18] include puncture site complications such as bleeding, pseudoaneurysm and arteriovenous fistula. Angioplasty site complications such as thrombosis, vessel rupture, dissection and distal emboli may occur. Systemic complications such as renal failure, myocardial infarction and cerebrovascular accidents are also known.

### Intra-arterial thrombolysis and mechanical thrombectomy

Traditionally, balloon embolectomy has been the treatment of choice for patients with an acute arterial embolism. However, because surgical thrombectomy is less successful in acute on chronic limb ischemia, intra-arterial thrombolysis is an attractive alternative. Good results have been obtained with the use of intra-arterial urokinase and t-PA. The recent deluge of devices also shows promise when used concurrently with thrombolysis. The commonest used device continues to be a large diameter catheter for aspiration.

The primary objectives of catheter-directed thrombolysis are to dissolve the occluding thrombus, restore perfusion and identify the underlying cause of arterial or graft thrombosis, allowing definitive correction.[19] Contraindications may be absolute, relative major or minor.[20] The absolute contraindications are stroke, an active bleeding diathesis, gastrointestinal bleeding within ten days and neurosurgery within the previous three months and intracranial trauma within the previous 3 months. The relative major contraindications are cardiopulmonary resuscitation within the previous 10 days, major nonvascular surgery or trauma within the previous 10 days, uncontrolled hypertension of more than 180 mm Hg systolic and more than 110 mm Hg diastolic, puncture of a noncompressible vessel, recent eye surgery and the presence of intracranial tumors.

The infusion methods include stepwise infusion, when the catheter is progressively advanced with thrombolysis, continuous infusion, graded infusion, forced periodic (e.g. pulse-spray) infusion and pharmacomechanic thrombolysis. Complications include severe systemic or intracranial bleeding, retroperitoneal or intra-abdominal bleeding, macroscopic hematuria and gastrointestinal bleeding (overt or occult) are rare.

### Vascular radiation therapy

Radiation therapy can be divided into external and intravascular (temporary source or a permanent source). To date, no randomized trial with long-term follow-up after external beam radiation has been performed.

### Gene therapy

Therapeutic angiogenesis postulates the manipulation of a spontaneous healing response by supplementation of growth factors (like vascular endothelial growth factor-VEGF) or transplantation of vascular progenitor cells. These supplements are intended to aid in the formation of arterial collaterals and promote the regeneration of damaged tissues.

### Cryoplasty

Cryoplasty balloons in addition to mechanical remodeling of the stenotic lesion, reduce the temperature of the surrounding tissue to −10°C resulting in apoptosis of smooth muscle thereby reducing restenosis rates. Some published data show reduction in restenosis by 82% at the end of one year.[21]

### Techniques for revascularization in nonatherosclerotic occlusions

Fibromuscular dysplasia (FMD) is an arteriopathy of unknown origin. Angiography demonstrates a typical “string-of-beads” appearance. PTA has a success rate of between 82-100%, with a restenosis rate of 10-15%.[22] Takayasu's arteritis is a chronic inflammatory arteritis that affects large vessels. Endovascular treatment should be carried out in the quiescent phase. The technical success rates of PTA approach 95%[23] with the introduction of cutting balloons. Stents are used sparingly and as bail-out procedures since in-stent stenosis is extremely high in this sub-group.

### Interventional techniques in nonocclusive PVD

#### Stent grafts/covered stents:

**Figure 6 (A, B):**
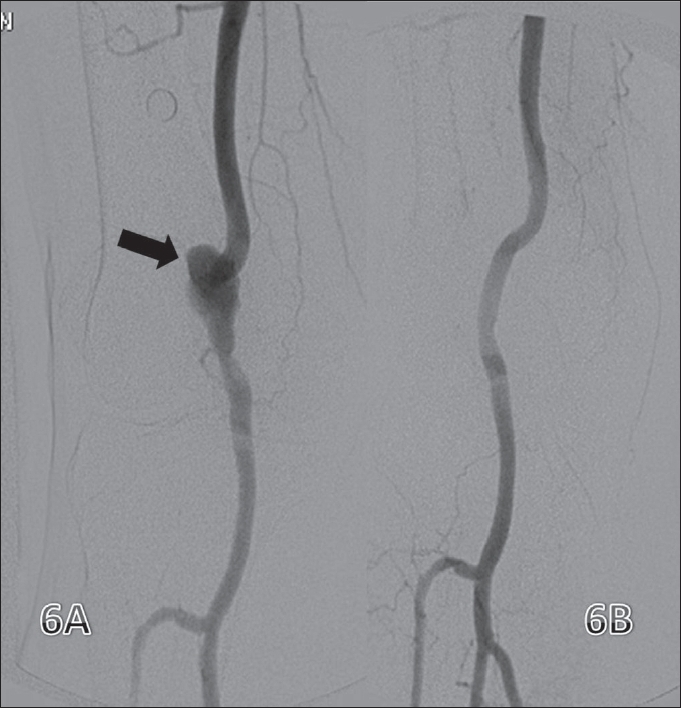
Popliteal artery aneurysm. Preintervention image shows a popliteal artery aneurysm (arrow). Post-intervention image (B) shows no filling of the aneurysm after deployment of a stent graft

Stent grafts are metallic stents with an impervious covering made either of Dacron or expanded polytetrafluoroethylene (ePTFE). A few manufacturers offer heparin coating to increase long-term patency rates. They are commonly used in thoracic and aortic aneurysms, traumatic arteriovenous fistulas (AVF), acute and chronic aortic dissections and pseudoaneurysms [Figure 6].

#### Abdominal aortic aneurysms:

The main principle of treatment whether by open surgery or endovascular access is to “exclude” the aneurysm from the circulation thus minimizing chances of rupture. In the absence of acute growth or symptoms, high-risk patients are seldom treated unless the size of the aneurysm exceeds 5.5 cm. Surgery has been associated with a 3-6% mortality rate and significant morbidity.[22] The idea of stent grafts for the treatment of aortic aneurysms, was first proposed by Parodi in 1991.[24] The use of endovascular techniques is justified in view of their significantly lower short-term morbidity,[22] but their long-term morbidity is still currently under study. In 70-80% of patients, the excluded aneurysm thromboses within a few hours of the procedure.[23] In the remaining, an endo-leak may occur which resolves in 50% of patients. The long-term outcomes of stent grafts are not known but delayed rupture has been reported.[25]

## Techniques Resulting in Vascular Occlusion

Embolization is defined as the deliberate occlusion of vessels for a therapeutic response. The choice of agent used also varies depending upon the size of the vessel. Temporary agents include gelfoam. Permanent agents may be either solid or liquid. Solid agents include polyvinyl alcohol (PVA), embolospheres, detachable fiber coils, platinum coils and detachable balloons. Liquid agents include absolute alcohol, N-butyl cyanoacrylate (NBCA) glue and Onyx (ethylene vinyl alcohol copolymer).

**Figure 7 (A-C):**
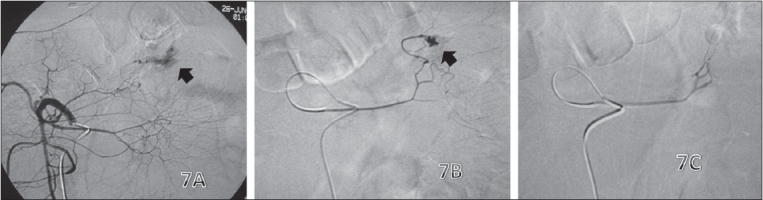
Gastrointestinal (GI) bleed. Angiogram (A) shows an abnormal vascular blush (arrow) from the branches of the superior mesenteric artery. Superselective catheterization and angiogram (B) of the culprit vessel confirms the finding of an active bleed (arrow). Post-embolization image (C) shows no evidence of the active bleed

The indications of vascular occlusion are (a) as a definitive procedure in arteriovenous malformation (AVM), hemostasis for bleeding (postpartum hemorrhage, trauma etc.) and chemoembolization; (b) as a palliative procedure in large AVMs, malignant masses and bleeding in chronic disorders and (c) as a preoperative procedure to reduce tumor vascularity. Embolization in acute bleeds [Figure 7] can prove to be a life-saving procedure and can be performed even in hemodynamically unstable patients, provided there has been a transient rise in blood pressure after rapid infusion of fluids and colloids.[26] The extremities have an extensive collateral network, so it is important trap the vessel to prevent retrograde filling of the injury site. Since precision is paramount in extremity vessels, coils are preferred. Temporary embolizing agents like gelfoam are advantageous in that all traumatic lesions eventually heal and the vessel may not need to be permanently sacrificed.

The management of vascular malformations depends on their type. In AVMs, the aim of endovascular treatment is to embolize and destroy the nidus. The best results are obtained with the use of liquid embolic agents such as Onyx and NBCA glue. Palliative therapy is performed with gelfoam soaked in absolute alcohol or a combination of glue and particles. Capillary hemangiomas usually involute spontaneously and conservative management may be initially attempted. Preoperative embolization may aid in surgical resection. Infants with high-output cardiac failure benefit from embolization therapy. Gelfoam pledgets, PVA particles or acrylic adhesives may be used. For AVFs, stent grafts, detachable balloons and multiple coils may be used. Venous angiomas are large blood-filled venous sacs, which may or may not be symptomatic. Patients often seek therapy for cosmetic purposes. The treatment involves injection of absolute alcohol in the venous sac under anesthesia. The alcohol is limited to 0.5 cc per kg body weight.

### Tumor embolization

Embolization is useful for many hypervascular neoplasms like chemodactomas, soft tissue sarcomas, hemangioblastomas, hemagiopericytomas, renal cell carcinomas and juvenile nasal angiofibromas. The aim of tumor embolization is to administer therapy that either aids in subsequent surgical resection or to provide primary tumor control as an alternative to surgery. For certain tumors, it may be combined with the local delivery of chemotherapeutic agents in which case it is known as chemoembolization.

### Summary

Interventional vascular radiology has emerged as a first-line therapy in the management of PVD. Early detection followed by endovascular treatment forms the basis of treatment of PVD. Interventional procedures whenever feasible are effective and significantly reduce morbidity with reduced hospital stay and in most situations are more cost-effective than surgery. Although several new forms of treatment are coming into vogue, their routine use needs to be substantiated with further research.
